# Selective Effects of Cold Atmospheric Plasma on Bone Sarcoma Cells and Human Osteoblasts

**DOI:** 10.3390/biomedicines11020601

**Published:** 2023-02-17

**Authors:** Andreas Nitsch, Konrad F. Sieb, Sara Qarqash, Janosch Schoon, Axel Ekkernkamp, Georgi I. Wassilew, Maya Niethard, Lyubomir Haralambiev

**Affiliations:** 1 Center for Orthopedics, Trauma Surgery and Rehabilitation Medicine, University Medicine Greifswald, Ferdinand-Sauerbruch-Straße, 17475 Greifswald, Germany; 2 Department of Trauma and Orthopaedic Surgery, BG Klinikum Unfallkrankenhaus Berlin, Warener Straße 7, 12683 Berlin, Germany; 3 Sarcoma Centre, HELIOS-Klinikum Berlin-Buch, Schwanebecker Chaussee 50, 13125 Berlin, Germany

**Keywords:** cold atmospheric plasma, human osteoblast cells, bone cancer, osteosarcoma cells, Ewing’s sarcoma, apoptosis, reactive oxygen species, hydrogen peroxide

## Abstract

Background: The use of cold atmospheric plasma (CAP) in oncology has been intensively investigated over the past 15 years as it inhibits the growth of many tumor cells. It is known that reactive oxidative species (ROS) produced in CAP are responsible for this effect. However, to translate the use of CAP into medical practice, it is essential to know how CAP treatment affects non-malignant cells. Thus, the current in vitro study deals with the effect of CAP on human bone cancer cells and human osteoblasts. Here, identical CAP treatment regimens were applied to the malignant and non-malignant bone cells and their impact was compared. Methods: Two different human bone cancer cell types, U2-OS (osteosarcoma) and A673 (Ewing’s sarcoma), and non-malignant primary osteoblasts (HOB) were used. The CAP treatment was performed with the clinically approved kINPen MED. After CAP treatment, growth kinetics and a viability assay were performed. For detecting apoptosis, a caspase-3/7 assay and a TUNEL assay were used. Accumulated ROS was measured in cell culture medium and intracellular. To investigate the influence of CAP on cell motility, a scratch assay was carried out. Results: The CAP treatment showed strong inhibition of cell growth and viability in bone cancer cells. Apoptotic processes were enhanced in the malignant cells. Osteoblasts showed a higher potential for ROS resistance in comparison to malignant cells. There was no difference in cell motility between benign and malignant cells following CAP treatment. Conclusions: Osteoblasts show better tolerance to CAP treatment, indicated by less affected viability compared to CAP-treated bone cancer cells. This points toward the selective effect of CAP on sarcoma cells and represents a further step toward the clinical application of CAP.

## 1. Introduction

Tumors affecting the skeleton are a great medical and economical challenge. Independent of their origin—primary bone cancer or metastases—these tumors destroy the bone structure and function [1,2,3]. The treatment and prognosis of bone tumors depend on the one hand on their entity, localization, and spread, on the other hand, on patient characteristics such as age, morbidity, etc. [4]. Currently, surgery for radical tumor excision, adjuvant and neoadjuvant chemotherapy, and radiation as a single therapy or their combinations are state of the art in bone cancer treatment [5,6,7]. Local treatments such as laser ablation, thermal coagulation [8], or cryosurgery [9] serve as additional methods; however, they are not sufficient to be used as the sole therapy. The current treatment methods, especially chemotherapy, have plenty of undesirable side effects such as changes in bone development, increased bone resorption, and thus an increased risk of bone fractures [10,11,12]. Alternative techniques for intraoperative bone cancer therapy are needed.

In the last 10 years, cold atmospheric plasma (CAP) was introduced as a promising local anti-cancer therapy [13]. CAP is an ionized gas that appears very suitable for use on human tissue due to its body-like temperature (approx. 40 °C). The biological effects of CAP are associated with the numerous reactive species, ions, free electrons, electromagnetic fields, and the low level of UV radiation it contains. The reactive oxygen and nitrogen species (RONS), such as H_2_O_2_ [14,15], NO_2_^–^  [16], and ONOO^−^ [17,18] generated in CAP are also thought to be responsible for the mechanisms of action of CAP treatment in malignant cells. The cytotoxic effects of CAP are related to an increase in intracellular ROS [19,20], DNA damage [21,22], the disabling of antioxidant defenses [23,24], but also a direct influence on the cell life cycle such as cell arrest [25,26,27] and apoptosis [28,29] or necrotic cell death [30]. In numerous types of cancer such as glioblastoma, ovarian [31,32], gastric [33], pancreatic [34,35,36], lung [37], or colorectal carcinoma [35] breast cancer [38,39,40], and melanoma [41,42]—which also preferentially metastasize to the bones [43,44]— but also in primary bone tumors such as osteosarcoma, chondrosarcoma, and Ewing’s sarcoma, the anti-cancer effect of CAP has been proven in vitro [45,46,47]. CAP also induced growth inhibition in bone sarcoma cells, the stimulation of cell apoptosis, and the impairment of cell membrane functions [45,46,47,48,49,50,51,52]. Previous studies indicate that the plasma source and dose can have apoptotic effects on cancer cells while not affecting healthy cells [53,54,55]. However, a comparison of malignant bone cells and primary non-malignant bone cells with regard to possible selective in vitro effects of CAP remains elusive. Thus, the aim of this study is to treat osteosarcoma cells, Ewing’s sarcoma cells, and primary human osteoblasts with CAP and to compare the in vitro effects with respect to ROS accumulation, cell viability, and apoptosis.

## 2. Materials and Methods

### 2.1. Cell Culture

Two different human bone sarcoma cell lines were used: U2-OS (Osteosarcoma; Cell Lines Service, Eppelheim, Germany) and A673 (Ewing’s sarcoma; American Type Culture Collection, Manassas, VA, USA). In addition, non-malignant bone cells were used: human osteoblasts HOB (PromoCell, Heidelberg, Germany). U2-OS and A673 cells were cultured in Dulbecco’s modified Eagle’s medium (DMEM) containing 1.0 g/L glucose, 10% fetal bovine serum, 1 mM sodium pyruvate, and 1% penicillin/streptomycin (all reagents from PAN Biotech, Aidenbach, Germany). Human osteoblasts (HOB) were cultured in Human Osteoblast Growth Medium with Supplement Mix (HOBM; obtained from PromoCell, Heidelberg, Germany). All cells were incubated at 37 °C and 5% CO_2_.

### 2.2. Cold Atmospheric Plasma Treatment

CAP treatment was performed with the kIN-Pen^®^ MED plasma jet (Neoplas tools, Greifswald, Germany). The flow rate of the carrier gas argon was adjusted to 3 slm. Control cells were treated analogously but without igniting the gas plasma, i.e., only by argon gas flow. Cell suspensions were treated for different durations (specified in the corresponding methods section).

### 2.3. Growth Kinetics

In total, 2 × 10^3^ cells were suspended in 200 µL cell culture medium and were transferred to a 24-well plate and treated with CAP or carrier gas argon for 10 s. Immediately after the treatment, 800 μL cell culture medium was added to the wells. The cells were harvested with gentle trypsinization after 24, 48, 72, 96, and 120 h. The number of cells was determined using the CASY cell counter and analyzer model TT (OLS OMNI Life Science, Bremen, Germany). At least three independent experiments were performed.

### 2.4. Viability Assay

In total, 5 × 10^4^ (24 h and 48 h) or 1 × 10^4^ (120h) cells were suspended in 200 µL cell culture medium and were transferred to a 24-well plate and treated with CAP or carrier gas argon for 5 s, 10 s, and 20 s (24 h and 48 h) or 5 s, 10 s, 20 s, 30 s, and 60 s (120 h). The cell suspensions were transferred in 96-plate and incubated at 37 °C and 5% CO_2_ over 24 h, 48 h, or 120 h. Cell viability was determined using CellTiter-Blue^®^ Cell Viability Assay (Promega GmbH, Walldorf, Germany). The assay was carried out according to the manufacturer’s instructions [56,57]. The incubation period of the reagent was 1 h. The fluorescence intensity at 560/590 nm was recorded with a TECAN m200 multiplate reader (TECAN, Männedorf, Switzerland). At least three independent experiments were performed, each with triplicates. The fluorescence intensities of the CAP-treated samples were normalized to those of the carrier gas-treated controls.

### 2.5. Caspase-3/7-Assay

In total, 5.0 × 10^4^ cells were treated with CAP or argon for 10 s and incubated for 24 h and 48 h. To normalize the quantified fluorescence intensity to the cell number, a second plate was carried out parallel. After the incubation period, the used medium was removed and 100 μL of Caspase 3/7 detection solution DPBS with 2 µM CellEvent^TM^ Caspase 3/7 Green Detection Reagent (Thermo Fisher Scientific, Waltham, MA, USA) was incubated for 45 min. The fluorescence at 495/535 nm was recorded with a TECAN m200 multiplate reader (TECAN, Männedorf, Switzerland).

### 2.6. TUNEL-Assay

In total, 5.0 × 10^4^ cells were treated with CAP or argon for 10 s and incubated for 24 h and 48 h. The TiterTACS™ Colorimetric Apoptosis Detection Kit (Trevigen, Gaithersburg, MD, USA) was used according to the manufacturer’s instructions [58]. Absorption at 450 nm was quantified using the Infinite M200 plate reader (Tecan, Männedorf, Switzerland). The absorption of the samples was normalized to cell numbers using a parallel second plate.

### 2.7. Live-Dead Staining

The cells were treated with CAP or carrier gas argon for 10 s and were transferred to 96-well plates. After 24 h incubation, cells were stained with a live/dead cell imaging kit (Thermo Fisher Scientific, Waltham, MA, USA).

### 2.8. Intracellular Oxidative Stress Quantification

**Figure 1:**
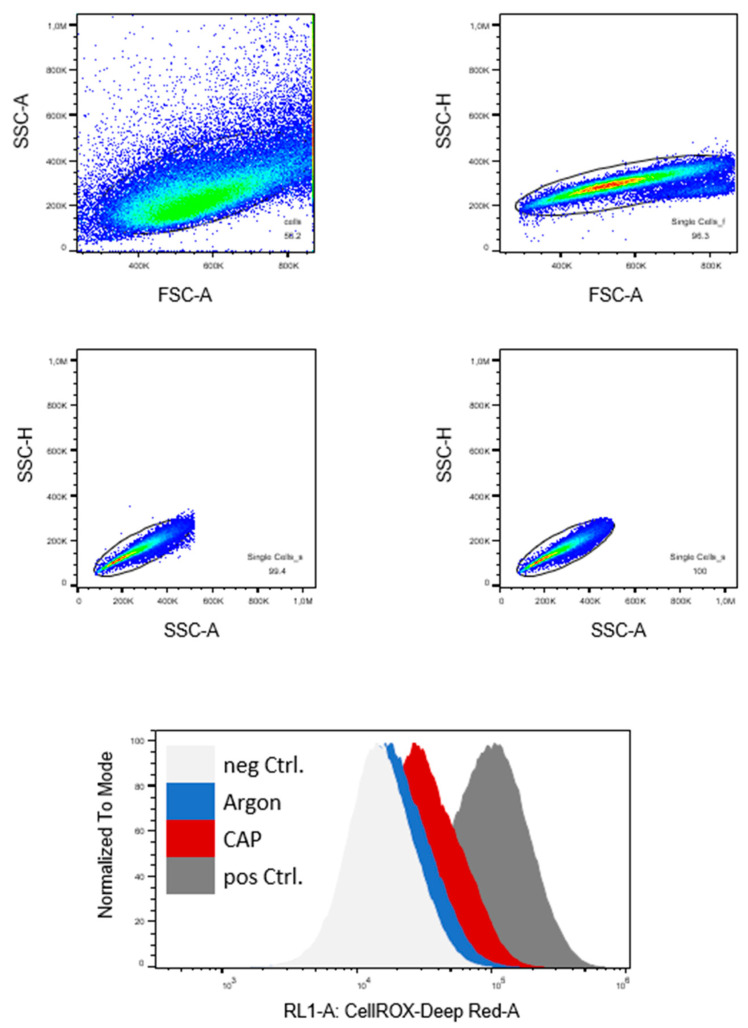
Human osteoblasts (HOB) and bone cancer cells (U2-OS and A673) were treated with cold atmospheric plasma (CAP) or carrier gas argon for 10 s. Controls were performed without treatment (neg Ctrl.) and with 5500 µm H_2_O_2_. Here, U2-OS is shown as a representative example. Gating strategy: Debris and doublets were excluded by forward- and side-scatter characteristics. The mean fluorescence intensity (MFI) of CellROX deep red was compared. SSC-A: side-scatter area, SSC-H: side-scatter height, FSC-W: forward-scatter width, FSC-H: forward-scatter height.

The cells were harvested, and the suspension was diluted to 1.0 × 10^6^ cells per milliliter in medium. The cell suspension (200 µL) was transferred in wells of a 24-well plate and treated with CAP or argon for 10 s. As a positive control, the cells were treated with 500 µM H_2_O_2_. After 1 h incubation, the cells were stained with CellROX deep red (Thermo Fisher Scientific, Waltham, MA, USA) and incubated for 30 min. After centrifugation, the labeled cells were resuspended in measuring buffer and analyzed in an Attunue^TM^ Flow Cytometer (Thermo Fisher Scientific, Waltham, MA, USA) and evaluated with FlowJo Software Version 10 (Tree Star Inc., Ashland, OR, USA). The gating strategy is shown in Figure 1. At least three independent experiments were performed. The mean fluorescence intensity (MFI) was normalized to the MFI of the argon-treated control cells.

### 2.9. Quantification of Hydrogen Peroxide Formation

The cells were harvested and diluted to 1.0 × 10^6^ cells per milliliter in HOBM und DMEM. Further, 200 µL cell suspension was treated with CAP for 0 s, 5 s, 10 s, 20 s, and 40 s. In addition, 200 µL of medium without cells was treated identically. Immediately after the treatment the medium or cell-free supernatant cells were transferred in a 96-well plate and diluted to 1:100, and the Amplex Red hydrogen peroxide assay (Thermo Fisher Scientific, Waltham, MA, USA) was carried out according to the manufacturer’s instructions [59]. After 6 h of incubation, the H_2_O_2_ concentration was quantified again.

### 2.10. Scratch-Assay

In total, 1 × 10^5^ cells were seeded in the wells of 2-well cell culture inserts (ibidi, Gräfeling, Germany) 24 h before the start of the assay. The insert was removed, and the cells were washed twice with DPBS, and 200 μL CAP- or argon-treated medium was added. To prevent the scratch from being closed by proliferation, the assays were performed under low serum conditions. With the software Zen 2012 pro, the cell-free area was recorded over 24 h in intervals of 2 h. The evaluation of the cell-free area was determined with ImageJ software. The quantified cell-free areas were normalized to the cell-free area at the beginning of the experiment.

### 2.11. Statistic

Unless otherwise stated, all data were depicted as mean values with standard deviation. At least three independent experiments were performed. The differences between the groups were evaluated using the t-test, ANOVA, and two-way ANOVA with a post hoc Tukey test. The software GraphPad Prism 9.1.2 was used for the evaluation and the graphic processing.

## 3. Results

### 3.1. Effects of Cell Growth and Viability

**Figure 2:**
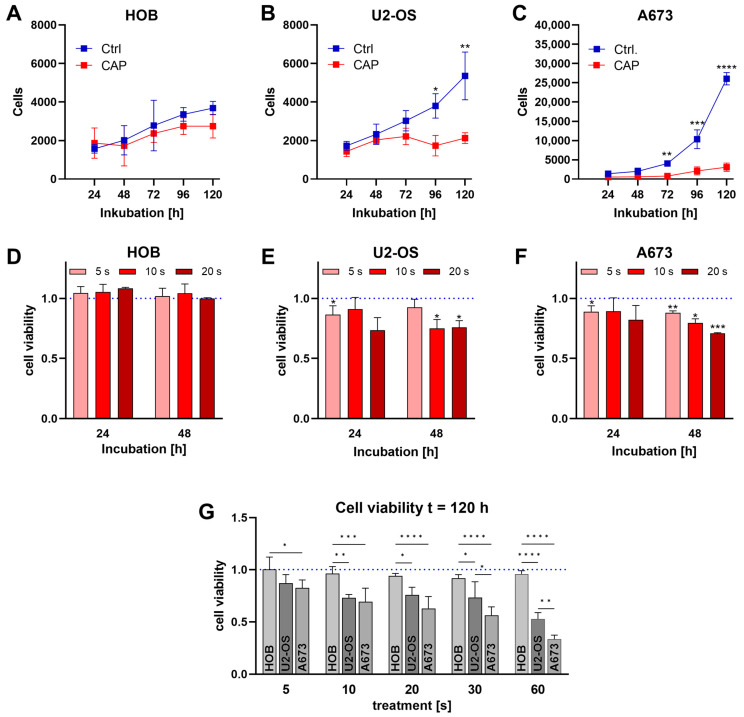
Human osteoblasts (HOB) and bone cancer cells (U2-OS and A673) were treated with cold atmospheric plasma (CAP). During the 120 h incubation, the cell count was determined every 24 h using a CASY Cell Counter and Analyzer (**A**–**C**). After CAP treatment, the non-malignant and the cancer cells were treated for 24 h, 48 h (**D**–**F**), and 120 h (**G**). Cell viability was determined using a titer blue assay. Mean values ± SD were normalized to the control treatments. Significant differences are indicated as follows * *p* < 0.05, ** *p* < 0.01, *** *p* < 0.001, **** *p* < 0.0001.

The cells were treated with CAP in a medium suspension for 10 s, to investigate the effects of CAP treatment on non-malignant human osteoblast and bone cancer cells. The number of viable cells was determined after 24 h, 48 h, 72 h, 96 h, and 120 h. Whereas cell proliferation of malignant cells is significantly inhibited after a single CAP treatment, the effects on non-malignant cells are less pronounced (Figure 2A–C). Complementary to the live cell count analysis, the cell viability was also examined. At 24 h and 48 h after treatment, the malignant cells showed a treatment time-dependent reduction in cell viability. In contrast, there was no reduction in cell viability in the non-malignant human osteoblast cells (Figure 2D–F). The different CAP treatment times were examined for up to 60 s, and the cells were incubated for up to 120 h. Compared to the control cells, treated with the carrier gas argon, the cell viability of the human osteoblasts was not significantly reduced by any of the tested CAP treatment times after 120 h. In contrast, a 10 s treatment in both malignant bone cancer cell lines led to a significant reduction in cell viability (Figure 2G).

Although the viability of HOB cells decreased slightly with increasing treatment duration, these effects were not as pronounced as they were for the malignant cell lines. The cell viability of the HOB is less impaired after 60 s CAP treatment than that of the two malignant bone cancer cell lines after 5 s of treatment.

Both the treatment time and the type of cells used had a significant impact on cell viability after 120 h incubation.

The viability of HOBs was less affected by CAP treatment and is significantly different from A673 cells after only 5 s and 10 s of treatment. The differences in cell viability between non-malignant and cancer cells increased with the prolongation of CAP treatment times (Figure 2G).

### 3.2. Induction von Apoptosis

**Figure 3:**
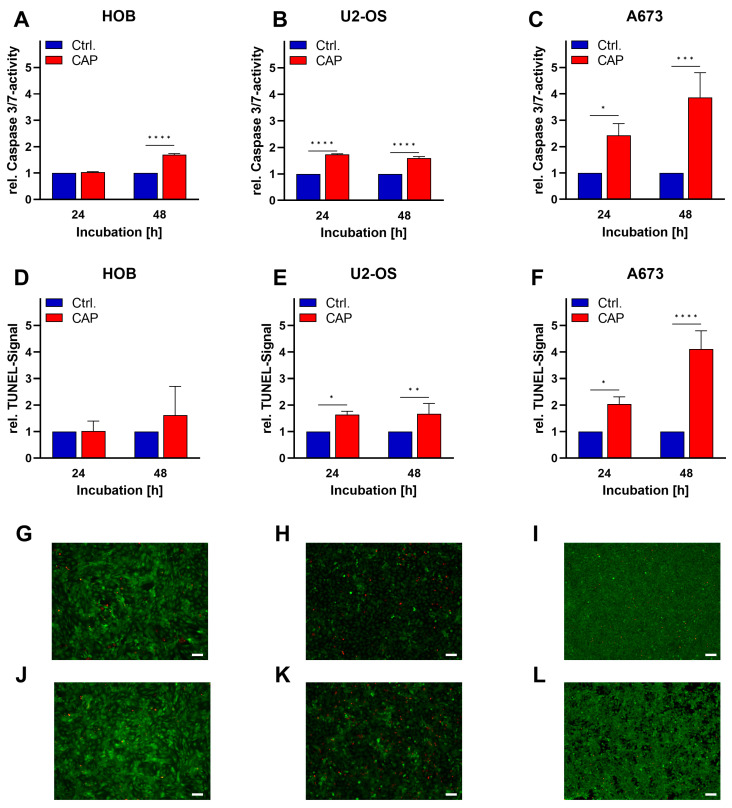
Human osteoblasts (HOB) and bone cancer cells (U2-OS and A673) were treated with cold atmospheric plasma (CAP) or argon for 10 s. After 24 h and 48 h, the apoptosis assays caspase-3/7 (**A**–**C**) and TUNEL (**D**–**F**) were performed. Cells were stained live (green)/dead (red) cell imaging kit (**G**–**L**), first row (**G**–**I**): argon treated, second row (**J**–**L**): CAP treated cells; representative images were shown. Data were given as mean ± SD of relative fluorescence (**A**–**C**) or absorption (**D**–**F**). Scale indicator is 100 µm. Significant differences are indicated as follows: * *p* < 0.05, ** *p* < 0.01, *** *p* < 0.001, **** *p* < 0.0001.

Furthermore, studies on apoptosis induction in the context of CAP were performed using Caspase-3/7 and TUNEL assays. At 24 h and 48 h after 10 s of CAP treatment, there was significantly increased caspase-3/7 activity in the bone cancer cell lines. In the U2-OS cells, the activity increased 1.5-fold compared to controls. In the A673 cells, the caspase-3/7 activity was increased 2.5-fold after 24 h and almost 4-fold after 48 h. No significant change in caspase activity was found in the human osteoblasts 24 h after treatment. After 48 h, an increase in caspase activity was also observed in the non-malignant cells (Figure 3A–C).

Apoptosis detection using the TUNEL method confirmed the results of the Caspase-3/7 assays. The malignant cells showed significantly increased TUNEL signals both after 24 h and after 48 h. Here, the effect was also more pronounced in the A673 in comparison with the other cells. Although after 48 h showed a tendency to increase the apoptosis rate, HOBs did not show any significant increase in the TUNEL signal (Figure 3D–E).

Live/dead staining confirmed that CAP treatments lead to a reduction in viability and an increase in dead cells. This effect was most evident in the A673 cell line, but no relevant difference was observed between the CAP-treated and the untreated cells in the HOB cells (Figure 3G–I).

### 3.3. Alteration of Extra- and Intracellular Oxidative Levels

**Figure 4:**
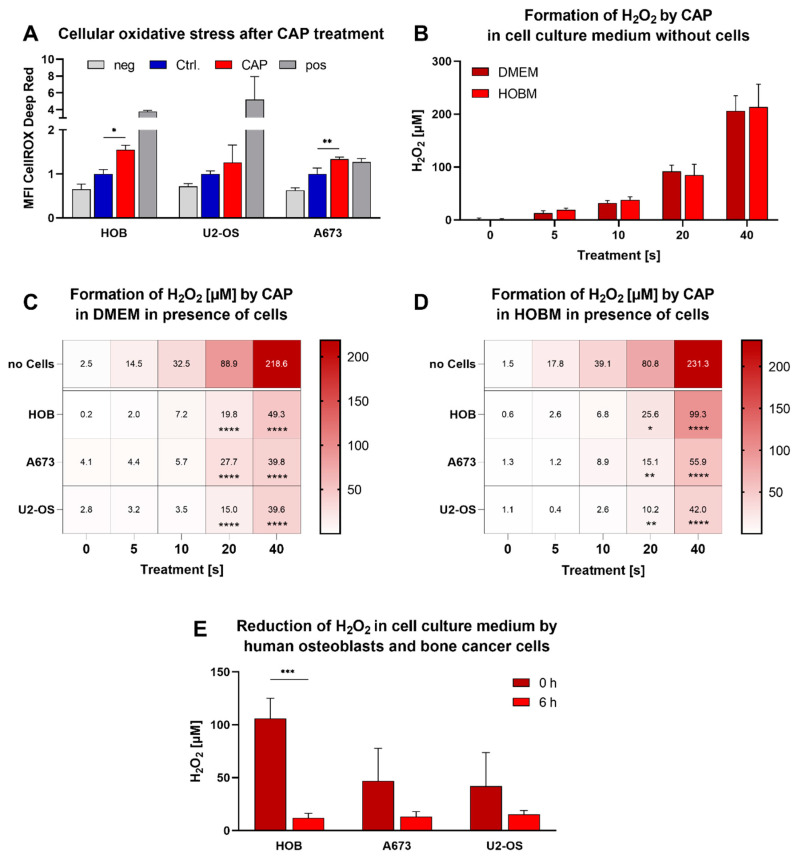
Intracellular oxidative stress increased significantly in human osteoblasts (HOB) and bone cancer cells (U2-OS and A673) after cold atmospheric plasma (CAP) treatment (**A**). Hydrogen peroxide (H_2_O_2_) was formed after CAP treatment. The amount of hydrogen peroxide formed differed depending on the treatment time, type of cell-free medium (DMEM and human osteoblast medium (HOBM) (**B**–**D**)) or cell suspension (**C**–**E**). Data were given as mean fluorescence intensity ± SD (**A**) or as mean concentration ± SD of H_2_O_2_ determined by a standard curve (**D**–**E**). Significant differences are indicated as follows: * *p* < 0.05, ** *p* < 0.01, *** *p* < 0.001, **** *p* < 0.0001.

One of the most important effects of CAP is the production of various reactive species. Therefore, the cellular oxidative stress after CAP exposure was investigated. CAP treatment led to an increase in oxidative stress in HOB and A673 (Figure 4A). The production of hydrogen peroxide by CAP was investigated by treating both cell culture media with CAP for up to 40 s. A treatment time-dependent formation of hydrogen peroxide was found. A longer treatment time generated a higher concentration of hydrogen peroxide (HOBM: r = 0.994, R^2^ = 0.988; DMEM: r = 0.995; R^2^ = 0.990). There was no significant difference between the two media types concerning the formation of hydrogen peroxide (Figure 4B).

Cell culture supernatants were analyzed to examine the buffering properties of the cells. The treatment time-dependent increase in hydrogen peroxide concentration by the cells was found to be <4-fold reduced (Figure 4C,D). This effect occurred in supernatants of all cell lines and all media used. To investigate the decomposition of hydrogen peroxide, the concentrations were quantified after 6 h of incubation. Here, the concentration declined in the supernatants of all cell lines (Figure 4E).

**Figure 5:**
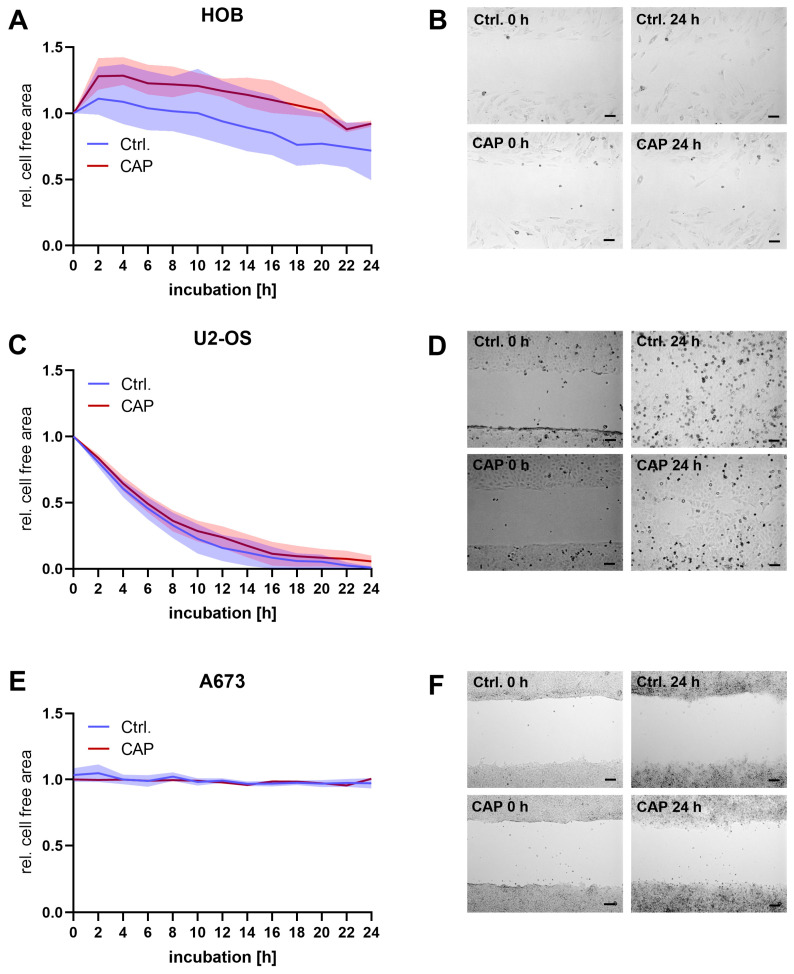
Human osteoblasts (HOB) and bone cancer cells (U2-OS and A673) were incubated in two-well cell culture inserts. After the removal of the insert, cells were treated with cold atmospheric plasma (CAP) or argon-treated medium. The cell-free area was recorded over 24 h in intervals of 2 h (**A**,**C**,**E**). The quantified cell-free areas were normalized to the cell-free area at the beginning of the experiment. (**B**,**D**,**F**): show representative images. Scale indicator is 100 µm. Data were shown as mean with range.

Wound healing assays were performed to investigate the influence of CAP treatment on cell motility. All cell types were slightly reduced in their motility following CAP treatment, although this effect was not statistically significant. In general, the baseline motility of the cells used differed considerably. Whereas the U2-OS cells had almost completely closed the cell-free area after 18 h, the A673 cells showed no tendency to move within 24 h (Figure 5).

## 4. Discussion

The current study was able to find clear differences in the cellular response of bone tumor cells and osteoblasts to the exposure of CAP. Although the CAP-induced formation of ROS was comparably high, the malignant cells showed a higher sensitivity to CAP.

Although many studies have discussed CAP as a promising anti-cancer therapy, its selectivity has rarely been reported [60,61,62,63]. In dermatological studies, an increased tolerance to the CAP exposure of skin fibroblasts (non-cancer skin cells) compared to melanoma cells was found. This differential susceptibility of non-malignant skin cells and melanoma cells to CAP exposure underscores the applicability of CAP in the clinical setting [64]. In this study, the main selective effects of CAP on malignant cells are attributed to ROS mechanisms. ROS has a strong impact on numerous biological processes in cells. In normal cells, ROS is controlled by regulation between the silver lining of low and high ROS concentration. ROS has a concentration-dependent influence on tumor cells and their environment. At moderate concentrations, signal cascades such as mitogen-activated protein kinase, c-Jun N-terminal kinase, and vascular endothelial growth factor (VEGF) are stimulated. At high concentrations of ROS, inhibitory effects on the angiogenesis, metastasis, and survival of cancer cells, primarily via the induction of apoptosis [65].

Altered survival signaling was indicated as the driving selective effect of CAP in glioblastoma cells [66]. The current study has shown that CAP treatment inhibits the growth of osteosarcoma and Ewing’s sarcoma cells confirming the findings of our previous studies [45,46,51], as well as those by other groups [48,67]. As CA-based reactive species are generated in the extracellular micro-environment, they not only specifically interact with the tumor cells but also with non-malignant cells, such as HOB. However, our in vitro study indicates that the non-malignant HOB shows lower susceptibility to CAP treatments. The loss of viability following CAP treatments is less pronounced in HOB in comparison to bone cancer cells. The possible selective effect of CAP in tumor cells versus non-malignant cells is believed to be due to their biological differences in terms of antioxidant resistance [68]. The production and metabolism of RONS by cancer cells are exploited therapeutically to unleash the effects of oxygen radicals. Thus, the increased ROS concentration exceeds the antioxidant resistance mechanisms of the cancer cell and leads to its apoptosis [68]. In the presented study, this hypothesis was confirmed by showing that apoptosis is enhanced in osteosarcoma and Ewing’s sarcoma in comparison to HOB.

The generation of ROS and its impact on the different cell types was quantified since the effects of CAP can be distinctively influenced by the type of cell culture medium [24,69]. Ingredients such as N-acetylcysteine, ascorbic acid, and other antioxidants can largely buffer ROS production by CAP and its effects [16,70,71,72]. Both DMEM and HOB medium showed a proportional increase in hydrogen peroxide depending on the CAP exposure time. The comparison of equivalent CAP treatment times in cell culture supernatant allowed for evaluating the individual level of ROS exposure. The quantification of the ROS levels 6 h after CAP exposure indicates cell type-specific differences regarding ROS tolerance. The non-malignant cells (HOB) showed a more pronounced tolerance to H_2_O_2_ than the bone cancer cell lines, although the osteoblasts were exposed to higher ROS concentrations. Although a high concentration of H_2_O_2_ is produced by CAP treatment, which is harmful to various cell types [73,74], in vitro studies show that the effects of CAP are not simply inducible by H_2_O_2_ treatment. Rather, the synergistic effect of numerous other reactive species such as NO_2_^−^/NO_3_^−^ together with hydrogen peroxide is held responsible for the cytotoxic and anti-proliferative effect in cancer cells [18,75,76,77].

The selectivity of the CAP treatment of osteosarcoma cells was previously reported by others [48]. Hamouda et al. [78] showed that HOB is more resistant to CAP treatment than to the osteosarcoma cell line SaOS-2. Two research groups have also compared the effects of CAP on mesenchymal stromal cells, another cell type from healthy bone, with the CAP effect on osteosarcoma cells. Mesenchymal stromal cells showed better CAP resistance than the bone cancer cells [48,79]. Ermakov et al. found not only a CAP-related increased proliferation activity but also an increased expression of osteogenic differentiation markers in human mesenchymal stem cells [79]. However, another study that focused on the effect of CAP at biocompatible doses on primary mesenchymal stromal cells of the bone marrow from various donors indicates no enhanced osteogenic differentiation potential [80].

Morphological changes in the surface and shape of numerous cancer cells after CAP treatment such as the loss of cytoplasmic protrusions and formation of tiny protuberances have been observed [81,82,83,84,85]. These changes are accompanied by architectural changes of the cytoskeleton such as F-actin [81,84,86], the reduced expression of integrin [81,87,88], and focal adhesion kinase [81], which is often related to reduced migration rates of CAP-treated cancer cells [87,89]. Changes in the cytoskeleton of bone cancer cells have been shown in our previous works [46,49]. The current results indicate a discrete reduction in cell motility; however, no differences were found between malignant and non-malignant bone cells.

Although the effects of CAP produced by different sources of osteosarcoma cells are comparable [51,90] the physical parameters such as voltage, current, power, and electromagnetic field are very individual for different plasma devices and have a direct influence on the plasma parameters and thus on the concentrations of the individual reactive species [62,91,92,93,94]. The quantification of the hydrogen peroxide concentration after different CAP exposure times clearly demonstrates a time-dependent increase. The CAP treatment is dosed over the exposure time and must be evaluated differently for each device and cell type [51,79]. Thus, it is necessary to perform studies investigating the effects of CAP from different sources on different bone cancer cells. The current study shows the effect of CAP not only on osteosarcoma cells but also on Ewing’s sarcoma cells in direct comparison to the effect on non-malignant bone cells.

## 5. Conclusions

The results of the presented study indicate a dose-dependent cytotoxic effect of CAP on osteosarcoma and Ewing’s sarcoma cells. The ROS-related apoptosis processes in the bone sarcoma cells appear to be largely absent in non-malignant HOBs. The HOBs show a significantly increased CAP resistance and thus better CAP tolerability. Our study provides important information on the possible selectivity of a CAP application for anti-cancer treatment in a pre-clinical in vitro setup.

## Data Availability

The data that support the findings of this study are available from the corresponding author upon reasonable request.
